# Long-term effectiveness of fremanezumab in episodic and chronic migraine patients in clinical routine – 24-months results from the prospective non-interventional FINESSE study

**DOI:** 10.1186/s10194-025-02259-x

**Published:** 2025-12-23

**Authors:** Andreas Straube, Gregor Broessner, Charly Gaul, Xenia Hamann, Axel Heinze, Torsten Kraya, Lars Neeb

**Affiliations:** 1 Department of Neurology, LMU Munich, University Hospital, Munich, Germany; 2 Department of Neurology, Innsbruck Medical University, Innsbruck, Austria; 3 Headache Center Frankfurt, Frankfurt, Germany; 4 Teva GmbH, Ulm, Germany; 5 Schmerzklinik Kiel, Migraine and Headache Center, Kiel, Germany; 6 Department of Neurology, Hospital Sankt Georg Leipzig gGmbH, Leipzig, Germany; 7 Department of Neurology, Headache Center Halle, University Hospital Halle, Halle (Saale), Germany; 8 Department of Neurology, Brandenburg Medical School Theodor Fontane, University Hospital Brandenburg/Havel, Brandenburg/Havel, Germany

**Keywords:** Calcitonin gene-related peptide, Fremanezumab, Migraine, Non-interventional, Real-world evidence

## Abstract

**Background:**

The CGRP pathway targeting antibody fremanezumab is indicated for prevention of migraine in adults with ≥4 migraine days/month. To address the limited availability in real-world long-term data, the FINESSE study was initiated to provide real-world evidence of long-term effectiveness of fremanezumab in clinical practice in an unselected migraine patient cohort.

**Methods:**

FINESSE was a non-interventional, prospective, multicentre, two-country (Germany, Austria) study observing migraine patients receiving fremanezumab over 24 months in clinical routine. The primary endpoint was the proportion of patients reaching ≥50% reduction in the monthly average number of migraine days (MMD) during the 6-month period following fremanezumab initiation. Secondary endpoints included changes from baseline in MMD, MIDAS and HIT-6 scores, and use of concomitant acute migraine medication. Exploratory endpoints comprised assessment of number and classes of concomitant preventive and acute migraine medications, and reduction in migraine severity. All secondary and exploratory outcomes were evaluated at multiple timepoints over the 24-month observation period. Safety data were obtained based on documentation of adverse events reported in normal clinical practice. Data analysis was performed using descriptive and, for comparisons to baseline, inferential statistics.

**Results:**

Data of 1016 patients (88.7% female; mean age 45.7 (SD 12.4); 55% episodic migraine; 45% chronic migraine) were evaluable in the full analysis set. Out of 987 patients in the primary endpoint set, the proportion of responders with a ≥ 50% MMD reduction during the 6-month period following fremanezumab initiation was 52.8% in all patients, 57.0% in episodic and 47.8% in chronic migraine patients. Further benefit was observed in terms of clinically meaningful MMD reductions from baseline, decrease in the use of concomitant acute medication, migraine severity, and improvements in disability scores, which were sustained over the 24-month observation period. No new safety signals were identified.

**Conclusions:**

Long-term fremanezumab treatment was associated with rapid, substantial and sustained improvement in both episodic and chronic migraine in a high proportion of patients in a real-world setting throughout the 24-month observation period. Real-world-data on tolerability corroborate the expected favourable safety profile of fremanezumab demonstrated in the pivotal clinical trials.

**Trial registration:**

The FINESSE study was retrospectively registered on the European Network of Centres for Pharmacoepidemiology and Pharmacovigilance (EUPAS44606) on December 8, 2021, and previously registered at Paul-Ehrlich-Institut (Federal Institute for Vaccines and biomedicines) on November 11, 2019.

**Graphical Abstract:**

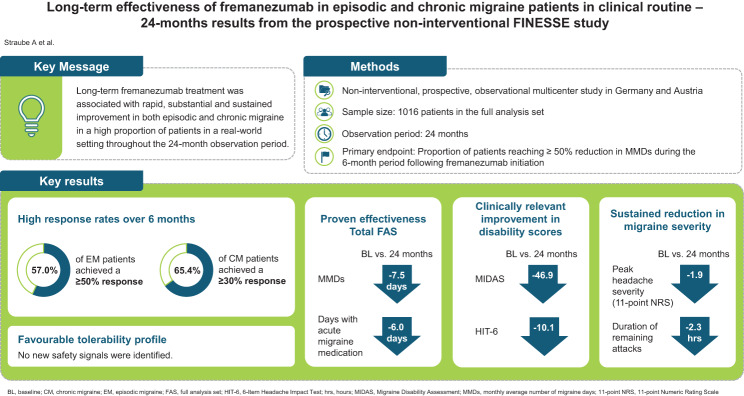

**Supplementary Information:**

The online version contains supplementary material available at 10.1186/s10194-025-02259-x.

## Background

Migraine is a common disabling neurological disorder affecting more than 1 billion people worldwide [1]. It is characterised by recurring headache attacks and associated symptoms such as nausea, vomiting, photophobia, phonophobia, and osmophobia. If left untreated, the duration of attacks varies between 4 and 72 hours [2]. Chronic Migraine (CM) is defined by the International Classification of Headache Disorders − 3^rd^ edition (ICHD-3) as headache occurring on 15 or more days/month for more than three months, which, on at least eight days/month, displays the features of migraine headache [3]. The main goals of preventive migraine treatment are to reduce the frequency, severity and duration of migraine attacks, to improve quality of life and to prevent medication overuse headaches (MOH) [2]. However, low adherence and tolerability of conventional migraine preventive medications, such as beta-blockers, antiepileptics, and tricyclic antidepressants, frequently lead to treatment discontinuation within the first 6 months [4, 5]. The discovery of the role of the calcitonin gene-related peptide (CGRP) in the pathophysiology of migraine [6–8] was a milestone in advancing migraine treatment and led to the development of specific monoclonal antibodies (mAbs) either targeting the CGRP ligand (galcanezumab, fremanezumab, eptinezumab) or the CGRP receptor (erenumab). All CGRP pathway targeting mAbs led to a reduction in the monthly average number of migraine days (MMD) comparable to conventional migraine prevention drugs [9–12] while offering advantages in safety, tolerability and low administration frequency [13–15]. Yet, due to pharmacoeconomic considerations, most healthcare policies in various countries require patients to have failed multiple attempts with conventional oral preventive drugs before reimbursement of CGRP pathway targeting therapies. As a result, the use of CGRP pathway targeting mAbs early in the treatment algorithm is limited by economic constraints even though these drugs have been demonstrated to be more effective when used after fewer prior therapies [16]. Indeed, effective long-term reduction of migraine days may have positive pharmacoeconomic consequences by improving quality of life, reducing absenteeism, decreasing comedication, and prevention of psychiatric comorbidity which is more likely in patients with higher migraine frequency [17, 18].

The CGRP pathway targeting mAb fremanezumab is indicated for the preventive treatment of migraine in adults who have at least 4 migraine days per month. It selectively targets the CGRP ligand and blocks both CGRP isoforms (α- and β-CGRP) from interacting with the CGRP receptor. Fremanezumab is highly specific for CGRP and does not bind to closely related neuropeptides [19]. Supported by robust evidence from pivotal trials demonstrating lower headache frequencies and reductions in acute headache medication use in the fremanezumab-treated groups compared to placebo [10, 20, 21], fremanezumab has become an established treatment option in clinical routine. In addition, fremanezumab was effective and well tolerated in patients with difficult-to-treat migraine who had failed up to four classes of migraine preventive medications [22], a common economical or health economic requirement for prescribing fremanezumab in many countries. Whereas a number of real-world evidence studies have confirmed the favourable benefit-risk profile, they had limitations regarding the number of enrolled patients, baseline migraine characteristics and/or duration of follow-up period [23–27]. Thus, the non-interventional study FINESSE was initiated to provide real-world evidence on long-term outcomes of fremanezumab treatment in clinical practice within a larger unselected migraine patient cohort.

## Methods

### Study design

FINESSE (Prospective observational study of fremanezumab effectiveness in chronic and episodic migraine patients in clinical routine) was a prospective, non-interventional, multicentre study conducted in Germany and Austria from November 2019 to January 2024. The study was approved by the ethics committee of the Ludwig-Maximilians-University Munich (Project No. 19–699) and registered on the European Network of Centres for Pharmacoepidemiology and Pharmacovigilance (EUPAS44606). FINESSE was designed, implemented and performed in accordance with the applicable legislation on non-interventional studies and the ethical principles set forth in the Declaration of Helsinki and the guidelines on Good Pharmacovigilance Practices. ICH-GCP (International Council for Harmonisation of Technical Requirements for Registration of Pharmaceuticals for Human Use - Good Clinical Practice) guidelines were followed. Patients were free to withdraw from the study at any time and for any reason. No diagnostic or therapeutic measures, exceeding the already necessary scope were required, and treatment routine was not altered by study participation. All patients provided their written informed consent prior to enrolment. Observation time per patient was 24 months, including assessments at baseline and, as per routine disease management, approximately every 3 months thereafter.

### Patients

Adult patients with EM or CM – diagnosed according to the criteria of the ICHD-3 [3] – who have been prescribed fremanezumab according to the Summary of Product Characteristics (SmPC) [19] as a treatment decision of their physician prior to study enrolment and who maintained a headache diary for at least 21 days within 28 days before fremanezumab initiation, were eligible for study inclusion. The diary ideally captured information on headache duration, headache severity, and headache characteristics. Following inclusion, patients continued clinical visits at the discretion of their treating physician as part of their routine management.

### Treatment

Patients were treated with fremanezumab monthly 225 mg or quarterly 675 mg (3 ×225 mg) based on their physician’s decision according to the SmPC with the first dose administered within 3 months (+7 days) of the day of enrolment. In accordance with the principles of a non-interventional study, concomitant acute or other preventive migraine treatments before study start were allowed to be continued, changed or unchanged throughout the study period.

### Study process

The baseline visit included obtaining written informed consent, assessment of patient eligibility, collection of baseline headache diary data, medical history and concomitant medications. If available, the migraine- and headache-related disability according to the patient-related outcome measures MIDAS (Migraine Disability Assessment) and HIT-6 (6-Item Headache Impact Test), as well as the emotional state according to the DASS (Depression Anxiety Stress Scales) questionnaire, were collected at baseline. The DASS consists of 3 patient-reported scales designed to measure the emotional states of depression, anxiety, and stress [28]. The MIDAS questionnaire is a 5-item instrument developed to assess migraine-related disability based on lost days of activity in 3 domains (work, household work, and non-work) over the previous 3 months. The MIDAS questionnaire has been shown to be reliable and valid for migraine, with substantially higher scores in migraine cases than non-migraine cases [29, 30]. In people with a baseline score > 20, a ≥ 30% reduction is considered clinically relevant [2]. The HIT-6 questionnaire has been shown to be a reliable and valid tool for the assessment of headache impact in patients with migraine [31]. It measures the adverse impact of headache on social functioning, role functioning, vitality, cognitive functioning, and psychological distress. A score reduction of ≥ 5 is considered clinically relevant [2]. At the follow-up visits, all headache diary data was checked for plausibility and consecutively documented including patient-reported outcomes as per MIDAS and HIT-6 questionnaires provided that patients completed these according to routine clinical practice. Safety data was recorded based on adverse events (AE) reported. All procedures performed within the scope of this study adhered to routine clinical practice.

### Endpoints

The primary endpoint was the proportion of patients reaching at least 50% reduction in MMD during the six-month period following the first dose of fremanezumab. Secondary endpoints included changes from baseline in MMD, in MIDAS and HIT-6 scores, and the use of concomitant acute migraine medication. Exploratory endpoints comprised assessment of number and classes of concomitant preventive and acute migraine medications, and reduction in migraine severity. MIDAS and HIT-6 scores were assessed at months 6, 12, and 24. All other secondary and exploratory outcomes were assessed at months 1, 3, 6, 12, and 24.

### Post hoc analyses

As obesity has been considered as a negative predictor of CGRP pathway targeting mAbs responsiveness in patients with CM [32], in a post hoc subgroup analysis, the change from baseline in MMD was evaluated for the subgroup of obese patients (BMI ≥ 30 kg/m^2^ [33]).

In order to exclude responder bias, post hoc sensitivity analyses of the changes in MMD and acute headache medication use were performed including all patients with available data at both baseline and month 24.

### Statistical analysis

Statistical analyses of the primary endpoint were conducted on the primary endpoint set (PES) presupposing the criterion of ≥ 4 migraine days within 21 documented days in the 28-day baseline period. All other effectiveness analyses were performed on the full analysis set (FAS) which included all patients in the safety analysis set (SAS) who had available diary entry data for the primary endpoint after treatment initiation. The SAS included all patients who received at least one dose of fremanezumab. All statistical analyses were of an explorative nature. Descriptive analysis of the data was performed using summary statistics for categorical and quantitative data. For continuous variables, statistic parameters including arithmetic mean, standard deviation, standard error of the mean and range were calculated. Frequency distributions for discrete variables were provided as percentage relative to the total sample. For comparisons to baseline, inferential statistics was used: The Wilcoxon signed-rank test was applied to test the differences between median baseline and the respective follow-up data. All tests were two-sided, and significance was declared at the 0.05 level. Baseline was defined as the 28-day period prior to fremanezumab treatment initiation. A sample size of approximately 1000 was considered sufficient as no significance testing was planned a priori and the study was set out to be purely descriptive. No statistical hypotheses were tested. All analyses were performed for the overall analysis population as well as stratified by migraine classification (EM versus CM). The statistical evaluation was conducted using the software package SAS release 9.4 (SAS Institute Inc., Cary, NC, USA).

## Results

**Fig. 1:**
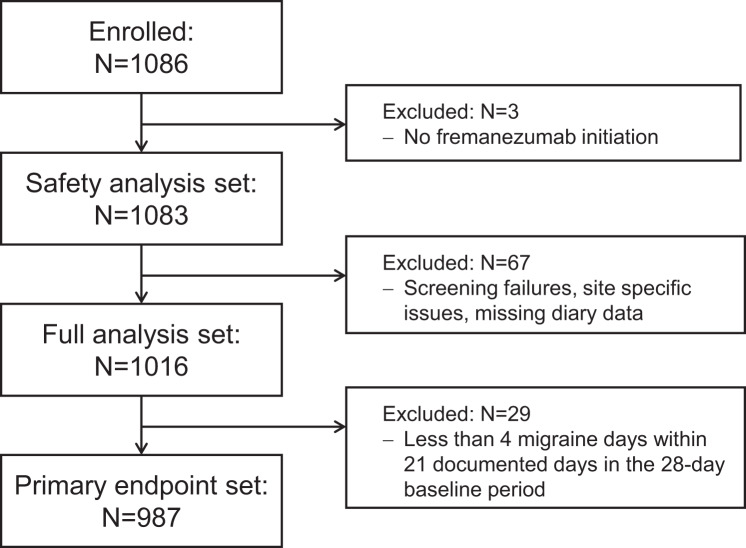
Flow chart of patients included

In total, 1086 patients were enrolled into the study at 123 sites in Germany and Austria from November 2019 to December 2021. Of these, three patients did not initiate fremanezumab and were excluded from the SAS (*N* = 1083). For effectiveness analyses, the FAS comprised 1016 patients after exclusion of 67 patients due to screening failure, site-specific issues or missing diary data. For analysis of the primary endpoint, 29 patients were excluded because they had less than four migraine days within 21 documented days in the 28-day baseline period, leaving 987 patients in the PES (Fig. 1). In terms of dosage, 906 patients (89.2%) exclusively received the monthly fremanezumab dosage while 11 patients (1.1%) received the quarterly dosage solely. Overall, 516 (50.8%) patients completed the study up to month 24. The main reasons (*n* > 10) provided for premature study discontinuation were loss to follow-up (12.2%), AE (11.1%), drug ineffective (6.4%), switch to another prophylactic migraine treatment (6.3%), withdrawn consent (4.6%), and insufficient compliance (2.2%). One patient ( < 0.1%) died due to COVID-19. The majority of patients were between 45 to < 55 years old (31.6%) and female (88.7%). A total of 559 patients (55.0%) were diagnosed with EM and 457 patients (45.0%) with CM. At baseline, 779 patients had completed the DASS questionnaire. A depression score of ≥ 10, an anxiety score of ≥ 6 and a stress score of ≥ 10 was documented in 188 (18.5%), 244 (24.0%), and 318 of patients (31.3%), respectively. The proportion of patients with a history of psychiatric diseases was 39.8%. In the 10 years prior to study entry, 98.0% of patients had received preventive migraine therapy, including treatment with another monoclonal antibody targeting the CGRP pathway in 168 patients (16.5%). Patient demographics and selected baseline characteristics are summarised in Table 1.

**Table 1:** Demographics and selected baseline characteristics (full analysis set, FAS)

Parameter	*N* = 1016
Sex, n (%)	
Female Male	901 (88.7) 115 (11.3)
Age (years), mean (SD)	45.7 (12.4)
BMI (kg/m^2^), mean (SD)	25.3 (5.0)
Type of migraine, n (%)	
Episodic migraine Chronic migraine	559 (55.0) 457 (45.0)
Alcohol consumption, n (%)	
Abstinent Irregulary Regulary Unknown	400 (39.4) 476 (46.9) 4 (0.4) 136 (13.4)
Smoking status, n (%)	
Non-smoker Former smoker Smoker Unknown	708 (69.7) 77 (7.6) 107 (10.5) 124 (12.2)
Time from initial migraine onset date to fremanezumab initiation (years), mean (SD), *N* = 759 Time from date of diagnosis to fremanezumab initiation (years), mean (SD), *N* = 963	26.0 (13.8) 15.2 (13.0)
Medical history, n (%)^a,b^	
None Psychiatric diseases Skeletal muscle, connective tissue and bone disorders Endocrine diseases Diseases of the nervous system Heart disease Surgical and medical interventions Diseases of the gastrointestinal tract Diseases of the respiratory tract, chest cavity and mediastinum Metabolism and nutritional disorders Vascular diseases Benign, malignant and non-specific neoplasms (incl. cysts and polyps)	247 (24.3) 404 (39.8) 183 (18.0) 166 (16.3) 146 (14.4) 114 (11.2) 85 (8.4) 78 (7.7) 78 (7.7) 64 (6.3) 58 (5.7) 54 (5.3)
Past preventive migraine therapy, n (%)^a^	
Patients with past preventive migraine therapy Antidepressants Anticonvulsants Beta-blockers Calcium Channel Blockers Onabotulinumtoxin A Any CGRP pathway targeting mAb Any Erenumab Erenumab 70 Erenumab 140 Galcanezumab	996 (98.0) 886 (87.2) 872 (85.8) 860 (84.6) 540 (53.1) 390 (38.4) 168 (16.5) 157 (15.5) 100 (9.8) 119 (11.7) 21 (2.1)
Duration of past preventive migraine therapy (months), mean (SD)^c^	
Antidepressants Anticonvulsants Beta-blockers Calcium Channel Blockers Onabulinumtoxin A Any Erenumab Erenumab 70 Erenumab 140 Galcanezumab	10.1 (18.8) 9.7 (19.5) 13.0 (22.2) 4.8 (9.7) 12.4 (18.5) 10.4 (8.5) 6.4 (6.5) 8.4 (6.8) 6.6 (4.0)
DASS^d^ at baseline, n (%); *N* = 779 (score missing: *N* = 237)	
Depression ≥10 Depression < 10 Anxiety ≥6 Anxiety < 6 Stress ≥10 Stress < 10	188 (18.5) 591 (58.2) 244 (24.0) 535 (52.7) 318 (31.3) 461 (45.4)
Mean (SD) number of monthly migraine days at baseline MIDAS Score at baseline, mean (SD) HIT-6 Score at baseline, mean (SD) Mean (SD) number of days with acute headache medication use at baseline Peak headache severity on the 11-point NRS at baseline, mean (SD) Mean (SD) duration of remaining attacks at baseline (h)	12.2 (6.1) 76.2 (64.5) 65.9 (4.6) 9.1 (5.2) 7.5 (1.9) 9.4 (6.2)

^a^Multiple answers possible

^b^Listed are diseases with a prevalence > 5%

^c^Durations for Erenumab 70 and Erenumab 140 were summed up

^d^Cut-offs according to [28, 34]

BMI, Body Mass Index; CGRP, Calcitonin Gene-Related Peptide; DASS, Depression Anxiety Stress Scales; HIT-6, 6-Item Headache Impact Test, MIDAS, Migraine Disability Assessment; 11-point NRS, 11-point Numeric Rating Scale; SD, Standard Deviation

### Treatment with fremanezumab

Patients initiated fremanezumab after a mean (SD) time of 26.0 (13.8) years following initial migraine onset and 15.2 (13.0) years following migraine diagnosis. Mean (SD) time from fremanezumab initiation to end of observation period was 17.7 (8.8) months.

### Effectiveness

**Fig. 2:**
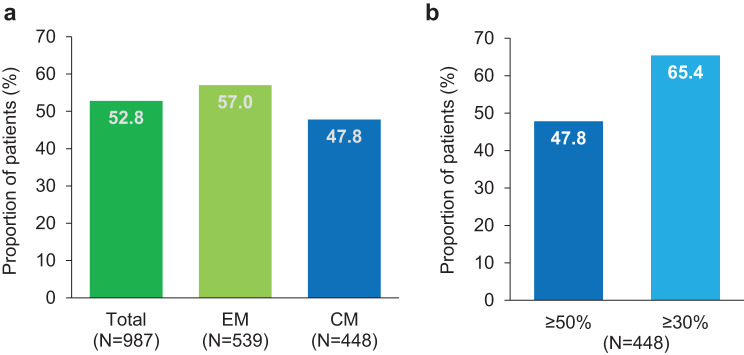
Proportion of patients with ≥ 50% reduction in MMD over 6 months versus baseline (**a**) and proportion of CM patients with ≥ 50% and ≥ 30% reduction in MMD over 6 months versus baseline (**b**) CM, chronic migraine; EM, episodic migraine; MMD, monthly average number of migraine days

**Fig. 3:**
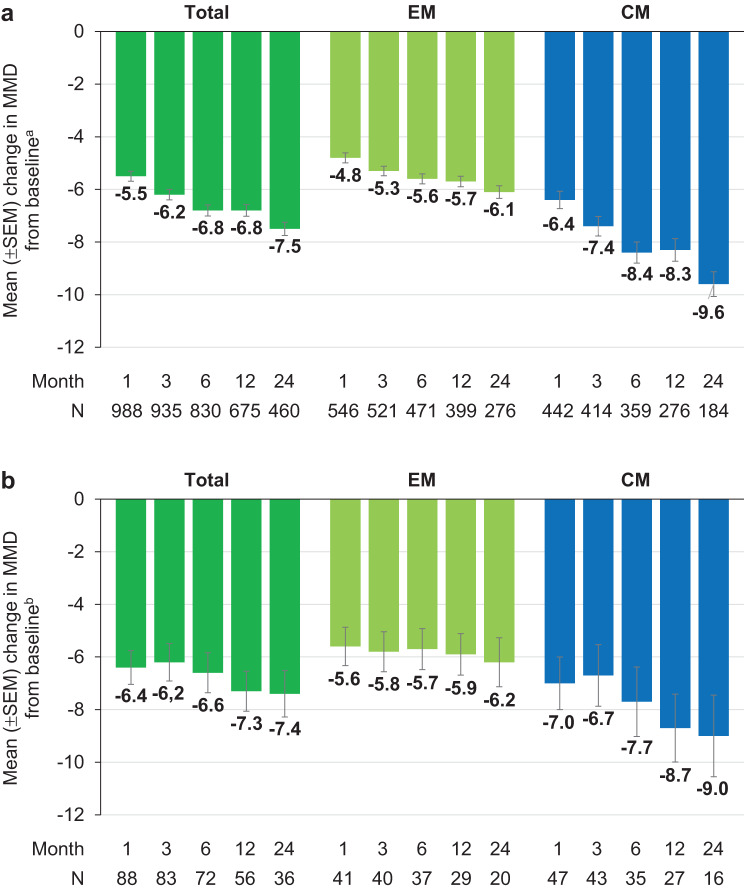
Mean change from baseline in MMD for total FAS (**a**) and obese subgroup (BMI ≥30 kg/m^2^ [33]) (**b**) ^a^MMD at baseline: Total = 12.2 (*N* = 1016); EM = 9.3 (*N* = 559); CM = 15.8 (*N* = 457) *p* < 0.0001 versus baseline (Wilcoxon test for paired samples performed at months 1, 3, 6, 12, and 24, two-sided p, difference is based on medians) ^b^MMD at baseline: Total = 13.0 (*N* = 91); EM = 8.9 (*N* = 41); CM = 16.4 (*N* = 50) *p* < 0.0001 versus baseline (Wilcoxon test for paired samples performed at months 1, 3, 6, 12, and 24, two-sided p, difference is based on medians) CM, chronic migraine; EM, episodic migraine; MMD, monthly average number of migraine days; SEM, standard error of the means

The proportion of patients reaching ≥ 50% reduction in MMD during the 6-month period after the first dose of fremanezumab (primary endpoint) was 52.8% for the total population, 57.0% for EM and 47.8% for CM (Fig. 2a). Among patients with CM, 65.4% achieved a ≥ 30% reduction in MMD over six months of treatment (Fig. 2b). Compared to baseline, the proportion of patients achieving an MMD reduction of ≥ 50% at months 1, 3, 6, 12, and 24 was 51.9% (525/1011), 57.0% (549/963), 60.9% (534/877), 59.5% (440/739) and 65.8% (339/515), respectively. The reduction in MMD from baseline increased significantly over time (post-hoc Wilcoxon signed-rank test for paired samples on medians) and was most pronounced in patients with CM (mean reduction by −9.6 days at month 24 versus baseline) (Fig. 3a). A post hoc sensitivity analysis including patients with available data at baseline and month 24 confirmed these results (Supplementary material 1: Supplementary Figure S1).

To exclude an effect of obesity, a post hoc analysis was performed in the subgroup of patients with BMI ≥ 30 kg/m^2^ (*N* = 91 at baseline). As reflected in MMD reduction, a significant difference to baseline was also found in this cohort of obese patients (Fig. 3b). With the exception of month 12 in the EM subgroup, no difference was found between the cohort of obese and the group of normal weight patients (BMI 18.5 - < 25 kg/m^2^, *N* = 310 at baseline) (EM: month 1: *p* = 0.1562, month 3: *p* = 0.1795, month 6: *p* = 0.1846, month 12: *p* = 0.0337, month 24: *p* = 0.2718; CM: month 1: *p* = 0.4639; month 3: *p* = 0.1060, month 6: *p* = 0.3275, month 12: *p* = 0.9247, month 24: *p* = 0.2630).

**Fig. 4:**
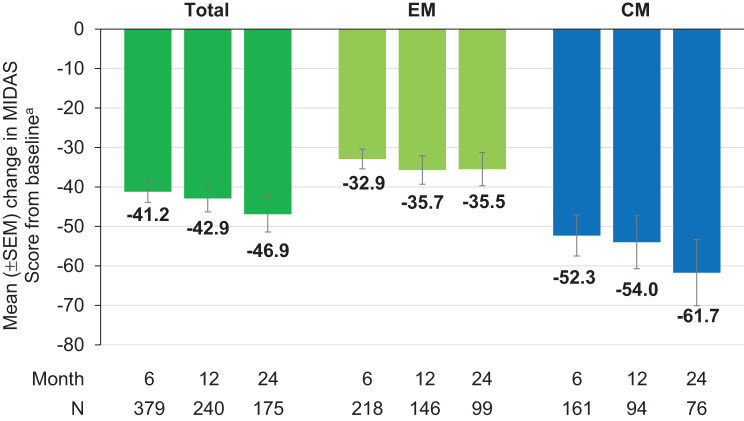
Mean change from baseline in the MIDAS score. ^a^Mean MIDAS score at baseline: Total = 76.2 (*N* = 608); EM = 59.2 (*N* = 333); CM = 96.7 (*N* = 275) *p* < 0.0001 versus baseline (Wilcoxon test for paired samples performed at months 6, 12, and 24, two-sided p, difference is based on medians) CM, chronic migraine; EM, episodic migraine; MIDAS, Migraine Disability Assessment; SEM, standard error of the mean

**Fig. 5:**
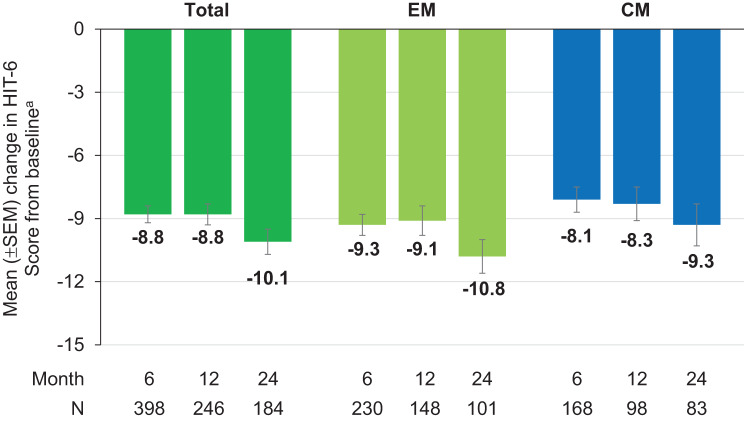
Mean change from baseline in the HIT-6 score. ^a^Mean HIT-6 score at baseline: Total = 65.9 (*N* = 644); EM = 65.5 (*N* = 346); CM = 66.4 (*N* = 298) *p* < 0.0001 versus baseline (Wilcoxon test for paired samples performed at months 6, 12, and 24, two-sided p, difference is based on medians) CM, chronic migraine; EM, episodic migraine; HIT-6, 6-Item Headache Impact Test; SEM, standard error of the mean

Assessment of migraine- and headache-related disability at months 6, 12, and 24, as measured by the MIDAS and HIT-6 questionnaires, respectively, both indicated a significant and sustained reduction in scores from baseline over 24 months in EM and CM patients as well as in the total population (Figs. 4 and 5).

**Fig. 6:**
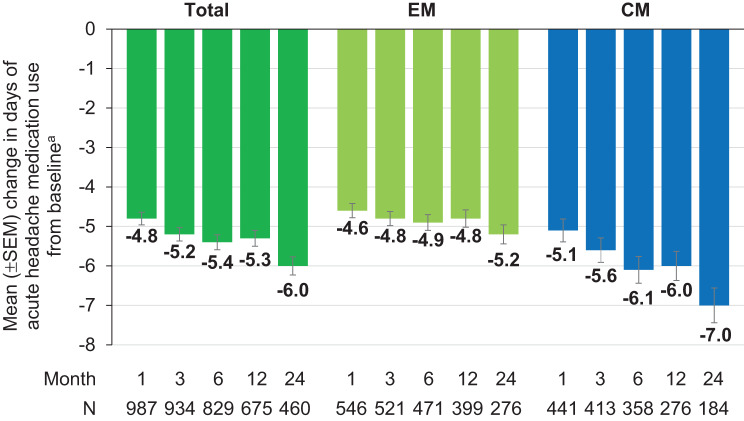
Mean change from baseline in the number of days of any acute headache medication use ^a^Average number of days with acute headache medication use at baseline: Total = 9.1 (*N* = 1015); EM = 7.8 (*N* = 559); CM = 10.7 (*N* = 456) *p* < 0.0001 versus baseline (Wilcoxon test for paired samples performed for months 1, 3, 6, 12, and 24, two-sided p, difference is based on medians) CM, chronic migraine; EM, episodic migraine; SEM, standard error of the mean

The monthly average number of days of any acute headache medication use decreased significantly over 24 months of fremanezumab treatment and was most pronounced in patients with CM (−7.0 days at month 24 versus baseline) (Fig. 6). A post hoc sensitivity analysis including only patients with available data at baseline and month 24 confirmed these results (Supplementary material 1: Supplementary Figure S2). In the total population, the mean (SD) monthly number of days with concomitant triptans declined from baseline [7.1 (5.13) days, *N* = 992] to month 1, 3, 6, 12, and 24 by −4.0 (4.54) [*N* = 965], −4.2 (4.59) [*N* = 916], −4.5 (4.72) [*N* = 811], −4.4 (4.73) [*N* = 662], and −4.8 (4.78) days [*N* = 453], respectively. In EM patients, a decline from baseline [6.4 (3.78) days, *N* = 545] by −4.0 (3.69) [*N* = 533], −4.1 (3.64) [*N* = 511], −4.3 (3.78) [*N* = 461], −4.2 (3.85) [*N* = 391], and −4.3 (3.95) [*N* = 270] days was observed, respectively. CM patients demonstrated a reduction in triptan use from 8.0 (6.29) days at baseline [*N* = 447] by −3.9 (5.41) [*N* = 432], −4.3 (5.57) [*N* = 405], −4.6 (5.72) [*N* = 350], −4.6 (5.77) [*N* = 271], and −5.5 (5.73) [*N* = 183] days, at the respective analysed months.

The number of patients taking concomitant preventive migraine medication was low and remained stable over 24 months (Supplementary material 2: Supplementary Table S1). In the total population of patients, antidepressants were used by fewer than 5.0% at all timepoints (47 patients at baseline and 20 patients at month 24), except 5.06% at month 3. The proportion of patients taking beta-blockers remained below 4.0% (26 patients at baseline and 18 patients at month 24), anticonvulsants 3.0% (21 patients at baseline and 10 patients at month 24), angiotensin II receptor antagonists 2.0% (13 patients at baseline and 7 patients at month 24), and calcium channel blockers and onabotulinumtoxin A below 1.0% (1 and 6 patients at baseline and 1 and 2 patients at month 24, respectively). Other concomitant preventive migraine medications outside these classes, were documented by fewer than 9.0% of patients (80 patients at baseline and 47 patients at month 24).

**Fig. 7:**
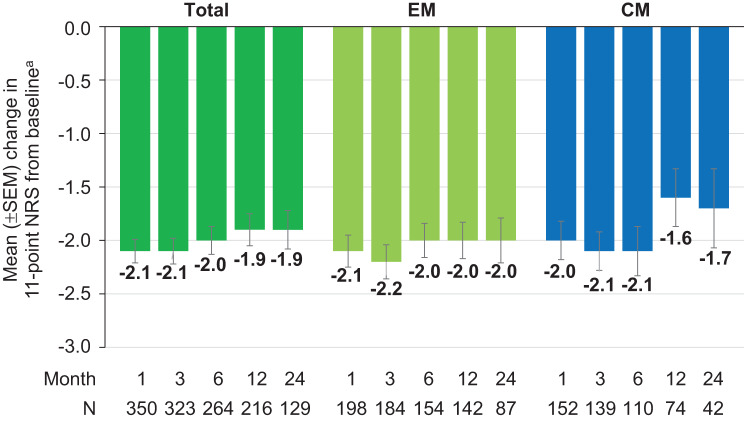
Mean change in the 11-point NRS for peak headache severity from baseline. ^a^Mean peak headache severity at baseline: Total = 7.5 (*N* = 465); EM = 7.5 (*N* = 269); CM = 7.5 (*N* = 196) CM, chronic migraine; EM, episodic migraine; 11-point NRS, 11-point numeric rating scale; SEM, standard error of the mean

**Fig. 8:**
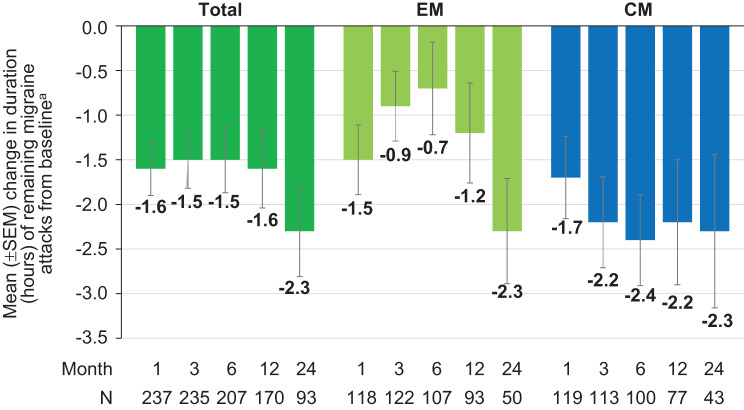
Mean change in duration of remaining attacks from baseline. ^a^Mean duration (hours) at baseline: Total = 9.4 (*N* = 292); EM = 7.9 (*N* = 157); CM = 11.1 (*N* = 135) CM, chronic migraine; EM, episodic migraine; SEM, standard error of the mean

Analysis of the 11-point Numeric Rating Scale (NRS) for the mean peak headache severity of remaining attacks revealed a stable and sustained reduction from baseline evident from month 1 through month 24 (Fig. 7). Data on peak headache severity assessed on a 4-point scale with none, mild, moderate, or severe pain at months 6, 12 and 24 were available for 163, 115 and 75 patients, respectively. An improvement in monthly peak headache severity was defined as a downward shift of at least one grade compared to baseline. This was achieved in 53.4%, 49.6% and 49.3% of patients at months 6, 12 and 24, respectively. Worsening, i.e. a shift upward of at least one grade, was observed in 6.7%, 3.5% and 5.3% of patients, respectively. A decrease as compared to baseline was observed in the mean duration (hours) of remaining migraine attacks at months 1, 3, 6, 12, and 24 (−2.3 hours at month 24 versus baseline) (Fig. 8).

### Tolerability

In total, 570 of 1083 patients in the SAS experienced 1511 adverse events (regardless of a causal relationship). The most frequently reported treatment-related AEs are summarised in Table 2. Eight patients experienced six serious AEs that were reported as related to fremanezumab: migraine (*N* = 3), myocardial infarction (*N* = 1), constipation (*N* = 1), reactive gastropathy (*N* = 1), cerebellar infarction (*N* = 1), and spinal subarachnoid haemorrhage (*N* = 1). All these patients with serious AEs had predisposing or confounding factors in their medical history and/or received concomitant medication.

**Table 2:** Most commonly reported related adverse events on patient level

**Related AE**^**a**^	N = 1083 (%)
Injection site erythema	76 (7.02)
Injection site pruritus	43 (3.97)
Injection site swelling	32 (2.95)
Constipation	30 (2.77)

^a^Relation ‘probable’ or ‘possible’; AE, adverse event

## Discussion

Real-World evidence of early and sustained (24 and 48 weeks) fremanezumab efficacy has been previously published [23, 35], however FINESSE is the first study reporting 24 months follow-up of continuous fremanezumab treatment in routine clinical practice. This study systemically assessed the effectiveness of fremanezumab in an unselected cohort of migraine patients under real-world conditions. Overall, the results demonstrate a rapid onset of effectiveness already within one month of treatment. Benefits were sustained over a period of 24 months as evident from the achieved reductions in multiple variables, including MMD, disability scores, peak headache severity, duration of remaining migraine attacks, and decrease in the concomitant use of acute migraine medications. Although effectiveness outcomes in terms of the primary and secondary endpoints are largely in line with those observed in fremanezumab randomised clinical trials [10, 20, 22, 36–39], results are slightly better in this real-world setting of clinical practice.

The primary endpoint on fremanezumab effectiveness in the FINESSE study was defined as the proportion of patients reaching at least 50% reduction in the monthly average number of migraine days during the 6-month period following fremanezumab initiation, a response considered clinically relevant in controlled trials of preventive treatment for migraine [2, 40]. This threshold was reached by more than half of EM patients (57%) and nearly half of those with CM (47.8%). In addition to a ≥ 50% reduction in MMD, the proportion of patients with CM achieving a reduction of 30% or more was evaluated, corresponding to the threshold considered to be effective in preventive migraine treatment in CM [2]. Within six months of fremanezumab treatment, this was achieved by almost two thirds of the CM patients (65.4%).

Almost all patients had a history of prior preventive migraine therapies (98.0%), including onabotulinumtoxin A (38.4%) and other CGRP pathway targeting mAbs (16.5%). The response rate is particularly remarkable, considering the subgroup of treatment refractory patients that, following a switch, still responded to fremanezumab treatment, as shown in a previously published FINESSE study subgroup analysis [41]. Thus, switching from other CGRP pathway targeting mAbs to fremanezumab may present a promising therapeutic option for patients experiencing inadequate efficacy or poor tolerability with prior other CGRP pathway targeting mAbs.

In line with results from the 52-week clinical trial [38], the reduction in MMD from baseline continued to increase over time. Likewise, a progressive increase in the proportion of ≥ 50% responders was demonstrated over three 12-month treatment cycles with mAbs targeting the CGRP pathway, predominantly (72.6%) erenumab [42]. Sensitivity analysis restricted to patients with data available at baseline and month 24 addressed attrition bias and confirmed the results as reflected in the FINESSE full analysis set. This observation aligns with previous real-world evidence of late ( > 3 to ≤ 6 months) and ultra-late response ( > 6 to ≤ 12 months) to CGRP pathway targeting mAbs in a proportion of patients [43, 44] and reinforces the guideline recommendation to treat patients with mAbs targeting the CGRP pathway for at least 12–18 months [45] or in cases of high-frequency episodic (≥8 MMD) or chronic migraine and concomitant diseases such as depression, anxiety disorder or chronic pain up to 24 months [46] before considering the possibility of treatment discontinuation.

Authors of an Italian study examining fremanezumab in a small real-world cohort of 90 patients over 12 months suggested a ceiling effect when sensitivity analyses using last observation carried forward (LOCF) to mitigate the influence of drop-outs showed no further significant improvement at months 6 and 12 of treatment [47]. Above mentioned sensitivity analysis to adjust for missing data due to drop-outs in FINESSE showed sustained improvements in MMD reduction up to 24 months of treatment and no such ceiling effect was observed.

In a multicentre cohort study, obesity emerged as a negative predictor of CGRP pathway targeting mAbs responsiveness in patients with CM [32]. Consequently, as the dosage of fremanezumab is not adapted to body weight, this could raise concern that patients with a higher BMI may be at disadvantage. However, the subgroup analysis of obese patients in FINESSE demonstrated that fremanezumab was equally effective in obese patients, with no difference in MMD reduction compared to patients with lower BMI, regardless of migraine frequency type. Due to the small sample size in the subgroups of obese CM patients in both studies (FINESSE, *N* = 50 at baseline; *N* = 16 at month 24; Italian Migraine Registry study, *N* = 32 [32]), results have to be interpreted with caution and verification in a larger sample is warranted. In addition, only 6% of patients in the Italian Migraine Registry study received fremanezumab [32]. Although there is also evidence that increased body weight is associated with increased erenumab clearance [48], data from the FINESSE study do not substantiate this finding for fremanezumab.

Profound and clinically relevant score reductions of ≥ 30% from baseline at assessed timepoints and regardless of migraine type were observed in migraine-related disability as measured by the MIDAS [2]. HIT-6 scores as a measure of headache-related disability decreased up to month 24 by more than 5 points as compared to baseline, which also qualifies as a clinically relevant improvement [2].

Furthermore, sustained reductions from baseline were also observed in the use of concomitant acute migraine medication. The effect was confirmed in a sensitivity analysis focussing on patients with available data at baseline and month 24 to rule out responder bias. The drop in concomitant use of acute medication, which is also seen in a 6-month US real-world study [18], occurs as early as one month after fremanezumab initiation and remains stable thereafter. The use of concomitant preventive migraine medication remained on a stable low level throughout the observation period. In consequence, the number of patients with changes in concomitant preventive migraine medication was too low to allow for assessing an impact on primary and secondary outcomes.

Real-world-data on tolerability are in line with the expected favourable safety profile of fremanezumab demonstrated in the previous randomised clinical trials [10, 20, 21], with no new safety findings emerging. Similar to the phase 3 RCT results [10, 20, 21], injection site reactions were the most common treatment-related AEs. Despite the known role of CGRP in the gastrointestinal tract [49], the occurrence of constipation was low, in line with other real-world evidence on treatment with fremanezumab [50].

The eligibility criteria in FINESSE have been selected to allow for a broad enrolment of patients that are usually underrepresented in randomised clinical trials in terms of previous and concomitant medication as well as comorbidities. In contrast to previously published real-world studies with smaller sample sizes and shorter follow-up [23–27], the FINESSE study enrolled 1086 patients from 123 sites in Germany and Austria who were supposed to be followed over 2 years with varied ages, medical history and history of past migraine treatment. Baseline demographics, such as age, sex, BMI, and prevalence of comorbidities were in line with known epidemiological data for migraine patients, where women are more often affected than men [51]. Many patients (69.1%) used concomitant medication other than preventive migraine medication and most (75.7%) had comorbid conditions in their medical history, reflecting the complexity of the migraine patient population in the real-world and underscoring the relevance of this study to everyday practice.

One limitation of this study is its non-controlled, real-world setting, in which results may be obscured by unknown confounders in patient cohorts. The absence of a comparator limits causal inference. Observed improvements could partly reflect spontaneous fluctuations in disease activity, or a placebo effect. Most importantly, the outcomes reported in patients’ headache diaries rely on the accuracy of the recorder and are subject to human error. Potential selection bias with regards to the selection of the participating investigational centres or individuals who consent to participate or who complete the study cannot be ruled out. This selection bias may affect the generalisability of the findings. Nevertheless, patients were selected according to a minimal set of eligibility criteria and treating physicians were asked to enrol consecutive patients whenever possible to maintain the observational character and enhance the external validity of the study. The drop-out rate of 49.2% between baseline and month 24 is a common issue in long-term non-interventional studies, which limits the representational quality of the patient cohort. However, patient attrition has been addressed by means of sensitivity analyses as mentioned above. Yet, a potential overestimation of long-term benefit due to attrition bias cannot be ruled out. Concomitant preventive migraine medications were allowed and could be continued or changed during the study period at the discretion of the treating physician. This flexibility mirrors real-world clinical practice but bears potential for confounding. However, as the number of patients taking concomitant preventive migraine medication was low and remained stable over the study period, the confounding influence is minimal. The potential impact of the COVID-19 pandemic on migraine symptoms has to be borne in mind when interpreting the results. For instance, COVID-19 may have influenced migraine frequency and severity through stress related exacerbations but also through potential reduction in migraine triggers due to workplace related stressors [52]. In addition, headache is a symptom of a COVID-19 infection as well as a common side effect of anti-COVID vaccines [53]. Although remote visits were feasible, restrictions imposed during the pandemic may have limited the number of visits to healthcare providers, which in turn may have impacted data completeness and treatment adherence. A strength of this study is represented by the presence of validated patient-reported outcome measures (MIDAS, HIT-6) to evaluate the impact on migraine- and headache-related disability [29–31]. Despite its limitations, the FINESSE study represents one of the largest prospective observational studies of fremanezumab, with a greater number of patients than the pivotal randomised clinical trials [10, 20, 22] providing supportive real-world evidence for its sustained and clinically meaningful effectiveness and favourable tolerability profile in routine clinical practice. Future research should address the effect of fremanezumab on MOH. Furthermore, the identification of independent predictors of treatment response may allow to identify patients who are particularly suited for the treatment with fremanezumab.

## Conclusions

Overall, long-term fremanezumab treatment was associated with rapid, substantial and sustained improvement in both EM and CM in a high proportion of patients in a real-world setting throughout the 24-month observation period. Benefit was observed in terms of clinically meaningful reductions in the average number of monthly migraine days, use of concomitant acute medication, peak headache severity, duration of remaining attacks, and improvements in disability scores. Real-world data on tolerability are in line with the expected favourable safety profile of fremanezumab demonstrated in the pivotal studies.

## Electronic supplementary material

Below is the link to the electronic supplementary material.

Supplementary Material 1

Supplementary Material 2

## Data Availability

The datasets generated during and analysed in the frame of this study are available from the corresponding author on reasonable request.

## Abbreviations

AE: adverse event
BMI: body mass index
CGRP: Calcitonin Gene-Related Peptide
CM: chronic migraine
DASS: Depression Anxiety Stress Scales
EM: episodic migraine
FAS: full analysis set
HIT-6: 6-Item Headache Impact Test
ICHD-3: International Classification of Headache Disorders − 3rd edition
ICH-GCP: International Council for Harmonisation of Technical Requirements for Registration of Pharmaceuticals for Human Use - Good Clinical Practice
LOCF: last observation carried forward
mAb: monoclonal antibody
MIDAS: Migraine Disability Assessment
MMD: monthly average number of migraine days
MOH: medication overuse headache
11-point NRS: 11-point Numeric Rating Scale
PES: primary endpoint set
RCT: randomised clinical trial
SAS: safety analysis set
SD: standard deviation
SEM: standard error of the mean
SmPC: Summary of Product Characteristics
