# Supplementary material for: Long-term effectiveness of fremanezumab in episodic and chronic migraine patients in clinical routine – 24-months results from the prospective non-interventional FINESSE study
Source: J Headache Pain. 2025 Dec 23;27(1):27. doi: 10.1186/s10194-025-02259-x (PMC12837042; doi:10.1186/s10194-025-02259-x)
Supplement: Supplementary file 1 — Supplementary Material 1 [file 10194_2025_2259_MOESM1_ESM.docx]

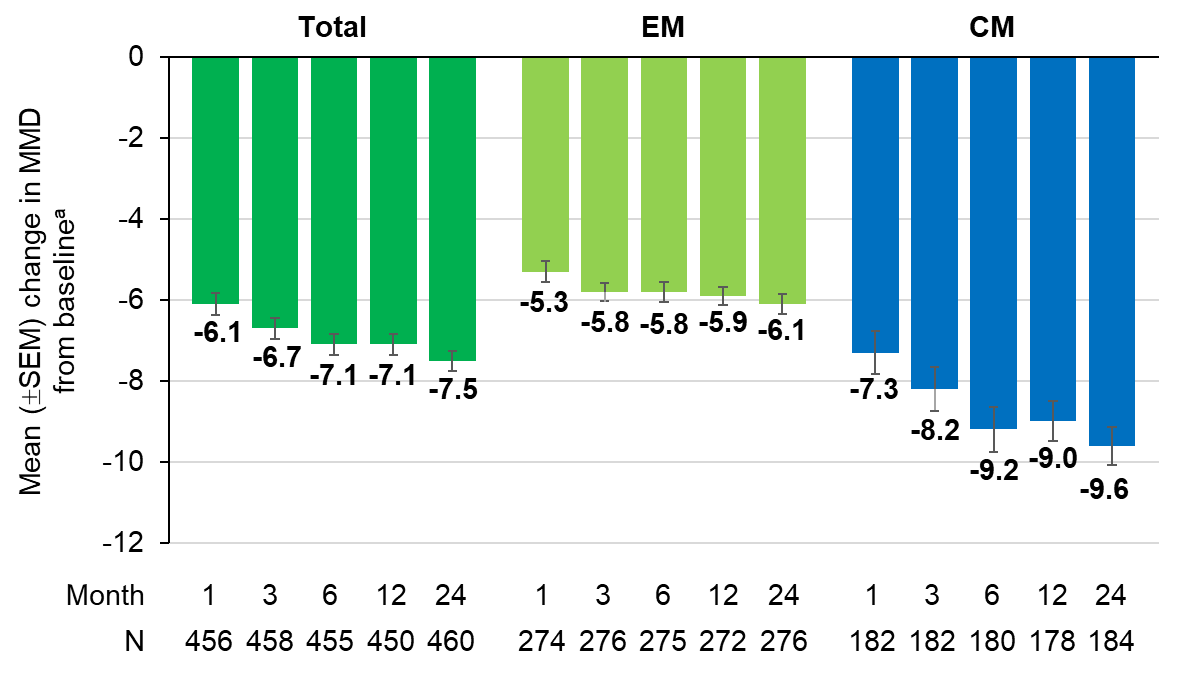


Supplementary Figure S1: Mean change from baseline in MMD for patients with data available at baseline and at month 24

^a^MMD at baseline: Total=11.3 (N=460); EM=9.2 (N=276); CM=14.4 (N=184)

p<0.001 versus baseline (Wilcoxon test for paired samples performed at months 1, 3, 6, 12, and 24, two-sided p, difference is based on medians)

CM, chronic migraine; EM, episodic migraine; MMD, monthly average number of migraine days; SEM, standard error of the means


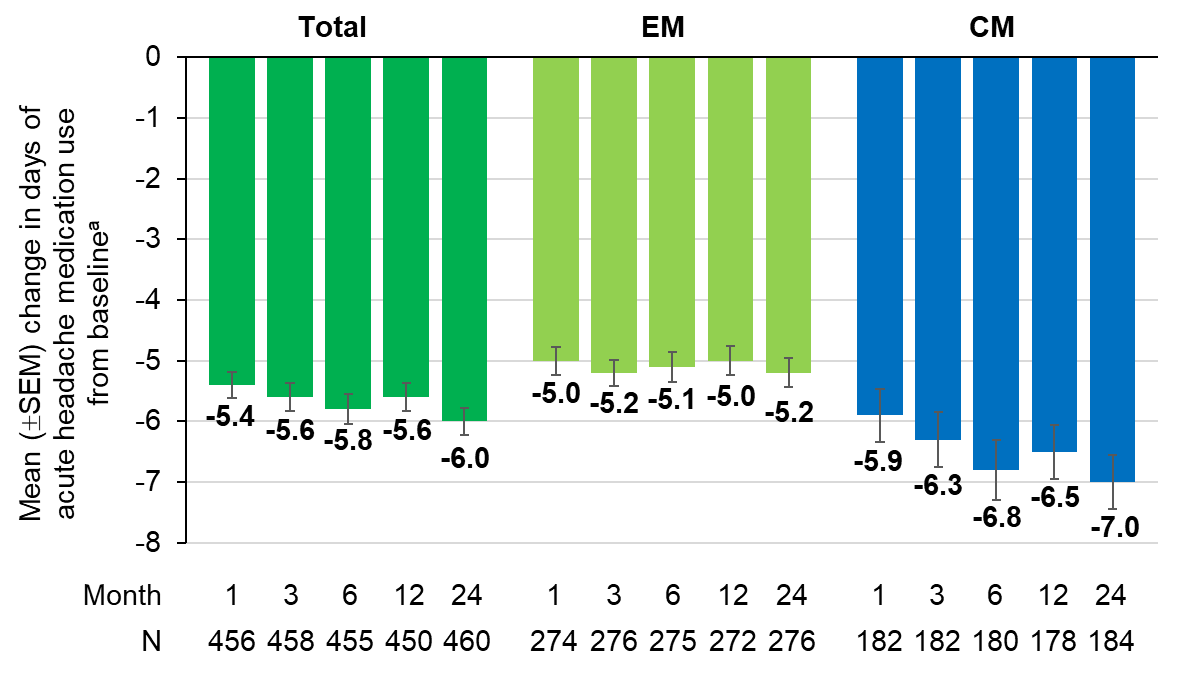


Supplementary Figure S2: Mean change from baseline in the number of days of any acute headache medication use for patients with data available at baseline and at month 24

^a^Average number of days with acute headache medication use at baseline: Total=8.9 (N= 460); EM=7.9 (N=276); CM=10.5 (N=184)

p<0.001 versus baseline (Wilcoxon test for paired samples performed for months 1, 3, 6, 12, and 24, two-sided p, difference is based on medians)

CM, chronic migraine; EM, episodic migraine; SEM, standard error of the mean
