# Supplementary material for: Long-term effectiveness of fremanezumab in episodic and chronic migraine patients in clinical routine – 24-months results from the prospective non-interventional FINESSE study
Source: J Headache Pain. 2025 Dec 23;27(1):27. doi: 10.1186/s10194-025-02259-x (PMC12837042; doi:10.1186/s10194-025-02259-x)
Supplement: Supplementary file 2 — Supplementary Material 2 [file 10194_2025_2259_MOESM2_ESM.docx]

Supplementary Table S1: Number (%) of patients taking concomitant preventive migraine medications at baseline and month 1, 3, 6, 12, and 24

|  | Baseline  n (%) | Month 1  n (%) | Month 3  n (%) | Month 6  n (%) | Month 12  n (%) | Month 24  n (%) |
| --- | --- | --- | --- | --- | --- | --- |
| Total patients | 1016 (100.00) | 1008 (100.00) | 989 (100.00) | 892 (100.00) | 727 (100.00) | 536 (100.00) |
| Angiotensin II  receptor antagonists | 13 (1.28) | 13 (1.29) | 12 (1.21) | 12 (1.35) | 7 (0.96) | 7 (1.31) |
| Anticonvulsants | 21 (2.07) | 23 (2.28) | 19 (1.92) | 15 (1.68) | 12 (1.65) | 10 (1.87) |
| Antidepressants | 47 (4.63) | 48 (4.76) | 50 (5.06) | 44 (4.93) | 27 (3.71) | 20 (3.73) |
| Beta-blockers | 26 (2.56) | 29 (2.88) | 26 (2.63) | 25 (2.80) | 21 (2.89) | 18 (3.36) |
| Calcium channel  blockers | 1 (0.10) | 1 (0.10) | 2 (0.20) | 2 (0.22) | 1 (0.14) | 1 (0.19) |
| Onabotulinumtoxin A | 6 (0.59) | 6 (0.60) | 4 (0.40) | 2 (0.22) | 1 (0.14) | 2 (0.37) |
| Other preventive  migraine medication | 80 (7.87) | 85 (8.43) | 83 (8.39) | 80 (8.97) | 62 (8.53) | 47 (8.77) |
